# Could violent conflict derail the London Declaration on NTDs?

**DOI:** 10.1371/journal.pntd.0006136

**Published:** 2018-04-19

**Authors:** Rebecca Y. Du, Jeffrey D. Stanaway, Peter J. Hotez

**Affiliations:** 1 Texas Children’s Hospital Center for Vaccine Development, Departments of Pediatrics and Molecular Virology and Microbiology, National School of Tropical Medicine, Baylor College of Medicine, Houston, Texas, United States of America; 2 Institute for Health Metrics and Evaluation, University of Washington, Seattle, Washington, United States of America; 3 Department of Biology, Baylor University, Waco, Texas, United States of America; 4 James A Baker III Institute, Rice University, Houston, Texas, United States of America; 5 Scowcroft Institute for International Affairs, Bush School of Public Policy and Public Service, College Station, Texas, United States of America; Emory University, UNITED STATES

The concept of the neglected tropical diseases (NTDs) is built around low socioeconomic status (SES) and poverty as the most important social determinants [1]. Poor health is not confined to poor people, but the burden of poor health is disproportionately greater within poor communities. A combination of insufficient social programs, unfair economic arrangements, and corrupt politics creates conditions that allow poverty to obstruct health [2]. Within this paradigm is the impact of violent conflict. Conflict not only facilitates the relationship between poverty and poor health, but it also is a social determinant of health in its own right. In other words, violent conflict enables poor health outcomes independent of poverty [3]. Here, we quantify the overlap among countries with conflict and countries with high prevalence of NTDs, and we discuss how violent conflict may undermine NTD control efforts, most notably for the 10 diseases slated for control, elimination, or eradication by the 2012 London Declaration on NTDs.

The 2012 London Declaration was created after the World Health Organization (WHO) 2020 Roadmap for eradication of NTDs established a priority for NTDs in public health and development efforts. In the London Declaration, 22 partners made a commitment to control, eliminate, or eradicate at least 10 NTDs by the year 2020. Partners included the United States Agency for International Development (USAID), United Kingdom Department for International Development (DFID), Bill & Melinda Gates Foundation, World Bank, and several pharmaceutical or medical technology organizations, including but not limited to Bayer, Merck KGaA, GlaxoSmithKline, Pfizer, and Eisai [4, 5]. This international agreement declared a commitment to “chart a new course toward health and sustainability among the world’s poorest communities to a stronger, healthier future” [4]. The London Declaration united leaders in global health and represented a redirection of global health and biotechnology capabilities toward addressing NTDs [5, 6].

## Methods

**Fig 1:**
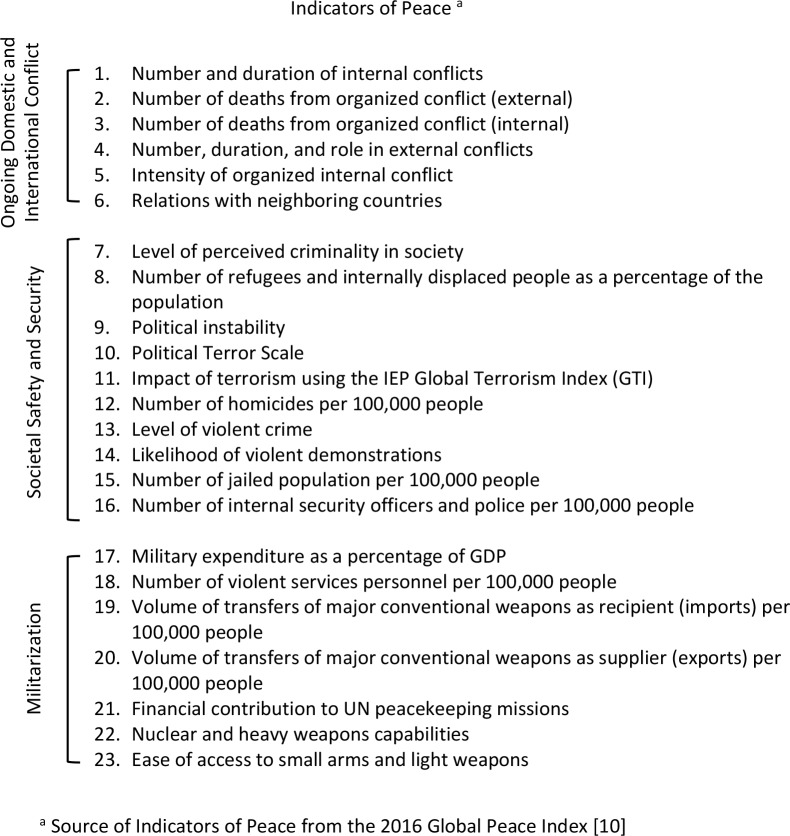
Twenty-three indicators used by the GPI [7] to determine levels of peacefulness in individual countries. GDP, gross domestic product; GPI, Global Peace Index; IEP, Institute for Economics and Peace.

Since 2007, the Institute for Economics and Peace (IEP) has ranked states and territories based on their “levels of peacefulness” in the Global Peace Index (GPI). In this annual index, peacefulness is measured using 23 indicators organized into three domains: ongoing domestic and international conflict, societal safety and security, and militarization (Fig 1) [7]. From composite scores, each country is designated a level of peace: very high, high, medium, low, or very low. Countries with “low” or “very low” levels of peace are described as countries at high risk for or currently experiencing conflict [7].

We identified countries with the highest prevalence of NTDs that were also determined by the GPI to have “low” or “very low” levels of peace. Data for the prevalence of NTDs were sourced from an analysis of the 2013 Global Burden of Diseases, Injuries, and Risk Factors Study (GBD 2013) [8]. The GBD systemically converges epidemiological data to quantify prevalence, morbidity, and mortality of 301 acute and chronic diseases and injuries in 188 countries [9]. Herricks et al. reviewed data concerning NTD burden in the GBD 2013 capstone papers and presented country-specific measurements for the prevalence of individual NTDs [8]. Prevalence in GBD assessments is defined as cases per 100,000 population. For data that are not available, the GBD used modeling approaches [8].

## Findings

Table 1 ranks countries with the greatest burden (defined by prevalence) of respective NTDs. Countries in column “1” have the highest prevalence of a specific NTD based on GBD 2013 [8]. All countries shaded in red have been identified by the GPI at least once between the years 2013 and 2016 to have “low” or “very low” levels of peace [7, 10–12].

**Table 1:** The presence of conflict among countries with the highest prevalence of specific NTDs.

	Countries with the Highest Prevalence of NTDs in 2013^a^		
Diseases	1	2	3
Schistosomiasis	Angola^b^	Gabon	Eritrea
Onchocerciasis	Liberia	South Sudan	Democratic Republic of the Congo
Human African Trypanosomiasis	Central African Republic	Democratic Republic of the Congo	South Sudan
Lymphatic Filariasis	Zambia	Eritrea	Gabon
Visceral Leishmaniasis	South Sudan	Sudan	Madagascar
Trachoma	Ethiopia	South Sudan	Mali
Cysticercosis	Burkina Faso	Peru	Liberia
Cutaneous Leishmaniasis	Afghanistan	Sudan	Syria
Chagas Disease	Bolivia	Argentina	El Salvador
Food-Borne Trematodiases	Laos	Thailand	China
Dengue^c^	Micronesia	Indonesia	Philippines
Trichuriasis	Kiribati	Marshall Islands	Jamaica
Hookworm	Papua New Guinea	Swaziland	Guatemala
Ascariasis	Malaysia	Equatorial Guinea	Afghanistan
Leprosy	South Sudan	Madagascar	Timor-Leste
Rabies^c^	Myanmar	Chad	Niger
Cystic Echinococcosis	Mongolia	Tajikistan	Zimbabwe
Other NTDs^d^	Afghanistan	Yemen	Senegal

Countries in red have been identified to have “low” or “very low” levels of peace by the GPI 2013–2016 [7, 10–12].

^a^ Source of prevalence of NTDs from Herricks et al. [8].

^b^Angola was identified to have a "very low" level of peace in 2007.

^c^For dengue and rabies, the table shows highest incidence rather than prevalence.

^d^ Other NTDs include dracunculiasis, relapsing fevers, typhus fever, spotted fever, Q fever, other rickettsioses, other mosquito-borne viral fevers, unspecified arthropod-borne viral fever, arenaviral hemorrhagic fever, toxoplasmosis, unspecified protozoal disease, taeniasis, diphyllobothriasis and sparganosis, other cestode infections, trichinellosis, strongyloidiasis, enterobiasis, and other helminthiases.

**Abbreviations:** GPI, Global Peace Index; NTD, neglected tropical disease.

Of the 18 NTDs (including “Other NTDs”) that were modeled separately for the GBD 2013, 15 were “highly endemic” in one or more countries identified to have “low” or “very low” levels of peace by the GPI 2013–2016. Highly endemic is defined as being ranked first, second, or third globally in terms of prevalence of the respective NTD. Only cysticercosis, trichuriasis, and hookworm were not highly endemic in one or more countries with high potential for or ongoing violent conflict. Six of the countries with the world's highest prevalence of a specific NTD were considered conflict nations, including the Central African Republic (CAR; human African trypanosomiasis), South Sudan (visceral leishmaniasis and leprosy), Ethiopia (trachoma), Afghanistan (cutaneous leishmaniasis), and Myanmar (rabies). For 4 of the NTDs, all 3 of the highest-prevalence countries were conflict nations. Dracunculiasis, also known as Guinea worm disease, is included in the “Other NTDs” category due to its near eradication—from January 1, 2016, to October 31, 2016, only 23 cases were reported. The only remaining endemic countries for dracunculiasis are Chad (15 cases in 2016), South Sudan (5 cases in 2016), Ethiopia (3 cases in 2016), and Mali (0 cases in 2016) [13]. Chad, South Sudan, and Ethiopia have all been identified by the GPI at least once between the years 2013 and 2016 to have “low” or “very low” levels of peace [7, 10–12].

Of note, several countries not shaded in red still carry scars from conflict that occurred prior to 2013. These include, but are not limited to, Angola [14], Timor-Leste [15], and Liberia [16]. Although not incorporated into our quantitative illustration, the impact of past conflict on NTDs in these countries should not be ignored. Additionally, with the exception of China and Thailand, all countries with “low” or “very low” levels of peace and high endemicity of a specific NTD were categorized by the most recent World Bank classification of economies as “low income” or “lower-middle income” [17]. This contrasts with the “blue marble health” concept that G20 countries contribute the greatest number of global NTD cases due to the overwhelming burden of NTDs on poor communities in these nations [6]. Prevalence of NTDs is often highest in low- or lower-middle-income countries; therefore, the overlap of conflict with NTD prevalence illustrated in this analysis also is most apparent in low- or lower-middle-income countries.

## The impact of violent conflict on achieving the 2012 London Declaration

The following diseases are the 10 NTDs targeted by the 2012 London Declaration: dracunculiasis (Guinea worm disease), lymphatic filariasis, leprosy, human African trypanosomiasis (sleeping sickness), trachoma, schistosomiasis, soil-transmitted helminth (STH) infections, chagas disease, visceral leishmaniasis, and onchocerciasis (river blindness) [4]. Every one of these diseases, with the exception of STHs, is highly endemic in one or more countries with high potential for or ongoing violent conflict, as defined by the GPI. STHs also are most likely impacted by conflict; however, the two STHs modeled separately by the GBD 2013 (hookworm and trichuriasis) did not show high endemicity in countries with conflict; therefore, we did not include them in our discussion. We do hypothesize that the same mechanisms that increase risk for NTDs during conflict apply to STHs as well.

We cannot quantify the impact of conflict on each NTD; however, conflict has most likely influenced disease prevalence or disease control and elimination efforts for each of these NTDs in one way or another. It is already reported that ongoing efforts to control onchocerciasis in CAR [18] and sleeping sickness in South Sudan and Uganda [19–21] are being disrupted by violent conflict. In the following section, we discuss the major mechanisms through which violent conflict may increase NTD risk.

## How violent conflict leads to infectious diseases

The mutually enforcing relationship between conflict and infectious disease is multifactorial and dynamic. Beyrer et al. described two principal paradigms through which conflict and NTDs interact: factors that increase susceptibility to disease and factors that increase exposure to disease [22]. Fürst et al. provided an additional conceptual framework for the direct and indirect impacts of conflict on health and wealth [23]. Using the frameworks provided by Beyrer et al. and Fürst et al., we briefly describe the major factors through which conflict increases susceptibility and exposure to NTDs, first independent of poverty and then through poverty-mediated mechanisms. Although there may be some intermediate factors that perpetuate NTDs specifically, by and large the forces during conflict that increase NTD burden are the same as those that increase other infectious diseases. The factors we discuss are framed with consideration of broader socioeconomic, ecological, cultural, and political paradigms.

### Damage to medical care and public health services

Violence destroys health infrastructure and kills or injures health professionals, sometimes as collateral damage and other times as strategic targets [24]. In 2016 alone, attacks carried out mostly by Syrian government and Russian forces destroyed 108 health facilities (i.e., hospitals, clinics, pharmacies, etc.) and killed 91 health professionals [24–27] in Syria and Iraq. Violent conflicts also lead to unstable and unsafe environments that cause migration of skilled labor (i.e., nurses, physicians, pharmacists, technicians, etc.) [28]. Additionally, destruction of transportation, communication, and electrical infrastructure limits the capacity of remaining healthcare facilities to provide adequate medical and public health services. In this vein, conflict dismantles NTD vector control and mass drug-administration programs [29]. Past examples include the derailment of sleeping sickness programs in Angola, Sudan, and Democratic Republic of the Congo by conflict following decolonization in the 1960s and 1970s [20, 22, 30].

### Damage to health-supporting infrastructure

Health-supporting infrastructure includes, but is not limited to, food and water distribution and safety, sanitation services (e.g., trash collection, sewage treatment), and housing. Loss of health-supporting infrastructure increases risk for enteric [31–34], nonenteric, zoonotic, and anthropod-borne diseases [35–39]. Additionally, inadequate or unsafe food and water undermines human health, and the resulting malnutrition increases susceptibility to NTDs [40].

### Damage to the environment

Violent conflict may damage the physical environment. Deforestation, destruction of wildlife habitats, pollution, and disruption of water and soil sources are examples [41]. These anthropogenic alterations of vector, host, and pathogen environments may increase pathogen transmission [42]. Deforestation and destruction of wildlife habitats in particular have been suggested as key drivers of recent NTD and infectious disease outbreaks, from malaria [43, 44] to Ebola [45–47].

### Forced population displacement

Forced population displacement is accompanied by decreased economic opportunity, decreased access to health services, poor housing conditions, and exposure to and introduction of novel pathogens [48]—all of which increase infectious disease risk [22, 49, 50]. In 2005, the leading cause of deaths among internally displaced persons (IDPs) in Greater Darfur, Sudan, was diarrheal disease; among under-5-year-olds, diarrheal disease accounted for about a third of deaths [51]. Cross-sectional studies in countries from Sierra Leone [52] to Sri Lanka [53] to Palestine [39] have found high prevalence of intestinal parasites among displaced persons. Additionally, in the ongoing conflict in Syria, forced migration is a key factor in the increased incidence of cutaneous leishmaniasis in Syria and its neighboring countries [54].

### Loss of government capacity

Conflict disrupts bureaucracy and is associated with less effective, less accountable, and less transparent governments [7]. A principal reason is the immense economic burden of conflict [55]. In 2015, conflict in Iraq was estimated to cost US$152.3 billion (31% of its GDP), and conflict in Syria was estimated to cost US$56.7 billion (42% of its GDP) [10]. The expense of warfare increases prevalence of NTDs by diverting resources away from social protection and public health programs [56]. Conflict and post-conflict societies are at risk of failing Sustainable Development Goals (SDGs) because they have decreased capacity to effectively implement development programs [7].

### Weapons

Rape, disruption of health services (as discussed earlier), and infectious diseases themselves have all been used as weapons of war [48]. Sexual violence has the potential to increase incidence of sexually transmitted diseases. The systemic rape of women and girls during the 1994 Rwandan genocide played an integral role in the ethnic cleansing of Tutsi communities and is theorized to have facilitated the spread of HIV/AIDs in the region [57], although epidemiological data supporting this do not yet exist [58]. Systemic rape theoretically can also increase risk for sexually transmitted NTDs and infectious diseases, such as Ebola and Zika. Additionally, infectious diseases themselves have been used as biological weapons (e.g., water supplies contaminated with microorganisms or infected animals, blankets laced with smallpox) since the start of warfare [55] and will continue to be a threat during conflict for the foreseeable future [59].

### Increased levels of poverty

Conflict begets poverty. Between 1981 and 2005, countries experiencing major violence had poverty rates 21 percentage points higher than countries with no violence [48]; as of 2011, no low-income, conflict-affected country had achieved even one Millennium Development Goal (MDG). Conflict increases poverty by limiting economic opportunity (e.g., work permit limitations), reducing access to basic health services, undermining education, destroying assets, and limiting government social protection programs [48]. These mechanisms conspire to increase economic disparity between areas with conflict and areas without conflict. As a well-known and powerful social determent of health [2], poverty leads to increased risk for infectious diseases secondary to many of the mechanisms already discussed, most notably poor housing conditions and limited access to health and health-supporting services (e.g., sanitation services, clean food and water).

Urbanization complicates the relationship between conflict, poverty, and NTDs. By the year 2050, the IEP estimates the global urban population will grow by 2.5 billion people, and nearly 1.9 billion will be in the countries that currently have low or very low levels of peace [7]. Even in peaceful countries, accelerated urbanization can outpace the ability of governments to implement sanitation services and to develop adequate housing, which increases risk for enteric NTDs and NTDs with anthropod-borne vectors, respectively [3, 60]. With the added burden of conflict, the effects of urbanization on incidence and prevalence of NTDs are even more profound. The confluence of poverty, conflict, and urbanization is central to the prevalence of NTDs and infectious diseases around the world.

## Concluding comments

Given the potential for conflict to slow or halt the WHO and London Declaration targets for NTDs, it is worth exploring a high-level summit around this topic. Given the multisectoral impact of conflict on NTDs, such an initiative would go beyond the health sector exclusively and should require peace-building and global health efforts across multiple UN agencies. Two principal types of initiatives should be incorporated into NTD treatment and surveillance efforts. The first is addressing NTD burden in unstable states and active conflict settings. Addressing NTDs in areas with conflict will be difficult to say the least, but past successes—such as the Mobile Obstetric Maternal Health Workers (MOM) Project to provide maternal-health services for IDPs in Burma [61]—are hopeful examples that it can be done. The use of military personnel in Afghanistan to initiate routine deworming of children is another example of how NTDs may be addressed in areas with conflict [62]. In general, military personnel and infrastructure may be an underutilized resource in combating NTDs in conflict settings. With the development of any global health or NTD initiative, however, is the caveat to respect local political and cultural sensitivities. Any sustainable global health intervention must observe community needs and circumstances in order to avoid undermining local autonomy.

The second type of initiatives are peace-building efforts. A key component of this could include organizing temporary cessations in hostilities to facilitate preventive chemotherapy and other NTD control measures. The landmark, 4-month-long “Guinea worm” ceasefire implemented by the Carter Center and UN agencies in 1995 is a potent example of how NTD control could be potentially released from war, and it provides a model for future similar successes [63]. Precedence for the inclusion of global health and NTD initiatives in security and peace efforts already exists. In 2010, the United States National Security Strategy stated, “We are also pursuing the goal of reducing the burden of malaria and tuberculosis and seeking the elimination of important neglected tropical diseases” [64]. In order to achieve the London Declaration and future goals on NTDs, NTD fields must reciprocally incorporate peace-building efforts into NTD treatment, surveillance, and control initiatives.

## Limitations

The primary limitation is accuracy of NTD prevalence and burden data. Collecting data from low-resource countries, especially if there is conflict, is often challenging; therefore, GBD 2013 data for many diseases, such as cysticercosis and cystic echinococcosis, relied heavily on modeling approaches to overcome gaps in data [8]. On a global level, the use of modeling approaches is most likely negligible; however, they may have an impact on country-level estimation of NTD burden. More in-depth and specific limitations to the GBD 2013 data can be found in Herricks et al. [8]. Additionally, several aspects of this paper require further consideration. Questions include the specific relationships between foreign-military occupation and NTDs or global health in general. Does military occupation perpetuate instability? And in this vein, what then are the conflicts of interest in the substantial amount of funding provided to global health efforts by military organizations? This paper cannot draw definitive conclusions on the causative or correlative relationships between conflict and NTDs. It is meant to begin a discussion of the potentially underestimated impact of conflict on the burden of NTDs and to highlight the importance of this topic for NTD and global health fields.
